# Clinical Implementation of an Artificial Intelligence Tool in the Detection and Management of Pneumothoraces in Patients With COVID-19

**DOI:** 10.7759/cureus.42509

**Published:** 2023-07-26

**Authors:** Joshua G Hunter, Jonathan D Pierce, Robert C Gilkeson, Kaustav Bera, Amit Gupta

**Affiliations:** 1 Radiology, Case Western Reserve University School of Medicine, Cleveland, USA; 2 Radiology, University Hospitals Cleveland Medical Center, Cleveland, USA

**Keywords:** pulmonary barotrauma, artificial intelligence (ai), pneumothorax (ptx), covid-19, coronavirus disease

## Abstract

In this report, we present a series involving critically ill patients with known coronavirus disease (COVID-19) infection where a portable X-ray machine equipped with artificial intelligence (AI) software aided in the urgent radiographic diagnosis of pneumothorax. These cases demonstrate how real-world clinical employment of AI tools capable of analyzing and prioritizing studies in the radiologist’s worklist can potentially lead to earlier detection of emergent findings like pneumothorax. The use of AI tools in this manner has the potential to both improve radiology workflow and add significant clinical value in managing critically ill patient populations, such as those with severe COVID-19 infection.

## Introduction

The world remains in the midst of the ongoing coronavirus disease 2019 (COVID-19) pandemic caused by the novel coronavirus, severe acute respiratory syndrome coronavirus 2 (SARS-CoV-2). First declared a global pandemic by the World Health Organization on March 11, 2020, more than 765 million cases of COVID-19 and 6.9 million deaths have been reported worldwide as of April 30, 2023 [1]. Despite several effective vaccination regimens, the rise of more virulent global variants has contributed to its continued incidence with over 2.7 million cases reported in the four weeks preceding April 30, 2023 [1].

Several cases of spontaneous pneumothorax in patients with COVID-19 have been reported in the literature [2-4]. Additionally, a 2020 study observed that patients with COVID-19 infection requiring invasive mechanical ventilation experienced a higher rate of barotrauma than patients with acute respiratory distress syndrome (ARDS) or patients without COVID-19 infection [5]. However, pneumothorax remains a relatively atypical respiratory finding in COVID-19, and hence clinical suspicion at the time of diagnosis is often low [6]. As such, pneumothorax is typically identified on routine follow-up (i.e., non-STAT) imaging exams that may only be read several hours after initial imaging acquisition. Timely diagnosis and subsequent treatment of pneumothorax are critical given the serious potential complications, most notably progression to tension pneumothorax [7]. The utilization of an AI tool for pneumothorax detection has shown promise in accelerating the diagnosis of pneumothorax [8]. This case series explores the application of a pneumothorax-detecting AI tool to accelerate the detection and diagnosis of pneumothorax in patients with known COVID-19 infection.

Several hospitalized patients with known COVID-19 infection underwent routine chest X-rays acquired with an AI-enabled portable X-ray scanner. This machine was able to automatically detect the presence of pneumothorax in these patients and generate an alert in the patient archiving and communication system (PACS), enabling prioritization of these studies for rapid review by the radiologist and ultimately expediting urgent intervention. This article highlights the utility of this AI tool in promoting prompt detection of a critical finding by radiologists, which not only improves radiology workflow but also has the potential to add significant clinical value in the management of critically ill COVID-19 patients.

## Case presentation

Three patients with laboratory-confirmed COVID-19 infection with imaging findings that raised concerns for pneumothorax detected on an AI-enabled portable radiography machine were retrospectively identified through a search of saved cases in PACS. All three patients were in the ICU setting when AI-enabled chest radiography was performed and their ages ranged from 23 to 76 years.

**Figure 1:**
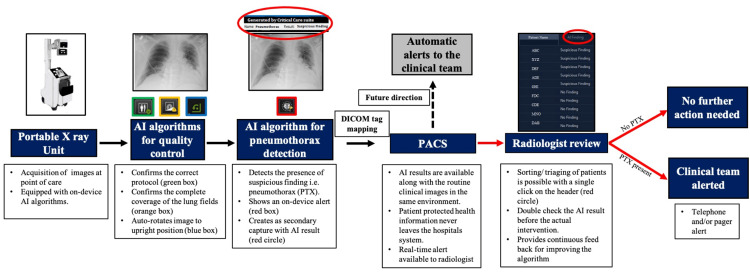
An illustration of the institutional workflow for clinical integration of AI-based pneumothorax algorithm Black arrows denote automated steps in the workflow. Red arrows denote manual steps in the workflow AI: artificial intelligence; PACS: patient archiving and communication system

Images were acquired on a portable radiography machine equipped with Critical Care Suite (GE HealthCare, Chicago, IL), an FDA-approved AI tool that can be used for pneumothorax detection [9]. This technology employs AI‐based image analysis algorithms to identify prespecified critical findings (e.g., pneumothorax) in frontal chest radiographs and flag the images in PACS to enable prioritized review by the radiologist. The AI tool also employs a sequence of three additional AI algorithms, which conduct automated quality checks to detect errors in the acquisition system and ensure that the input images are suitable for the pneumothorax detection algorithm. The integrated workflow involving this AI tool is depicted in Figure 1.

Case 1

**Figure 2:**
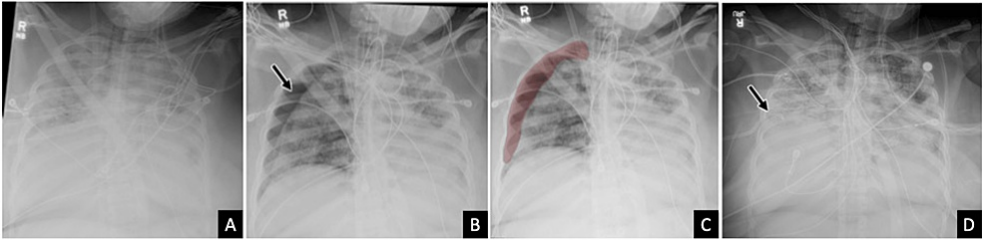
Chest radiographs of a 23-year-old pregnant female with worsening hypoxia and COVID-19 AP chest radiograph (A) demonstrates extensive diffuse bilateral airspace opacification, compatible with ARDS in the setting of COVID-19 pneumonia. Routine follow-up AP chest radiograph (B) demonstrates a new moderate-sized right pneumothorax (arrow), which was correctly flagged by the AI algorithm as shown in the associated heat map (C). The subsequent follow-up AP chest radiograph (D) after right chest tube placement (arrow) shows a near-complete resolution of the right pneumothorax AI: artificial intelligence; AP: anteroposterior; ARDS: acute respiratory distress syndrome; COVID-19: coronavirus disease 2019

A 23-year-old pregnant female with a past medical history of asthma presented to the emergency department (ED) with worsening shortness of breath, chest pain, fevers (maximum temperature of 101.3 °F), and poor appetite. Laboratory tests confirmed the diagnosis of COVID-19 and she was admitted to the Internal Medicine floor for further management. During hospitalization, she developed preeclampsia and her clinical picture continued to deteriorate. She required intubation and subsequently underwent emergent cesarian delivery, after which she was transferred to the ICU. Postoperatively, her course was complicated by multifocal pneumonia that quickly progressed to ARDS, requiring cannulation for veno-venous extracorporeal membrane oxygenation (Figure 2A). A follow-up, non-STAT, routine portable chest radiograph acquired on the morning of ICU day 16 was flagged in the PACS worklist by the AI tool as having a suspicious finding (Figures 2B, 2C). Subsequently, a new right apical and lateral pneumothorax was identified by the interpreting radiologist, and the primary team was notified within the hour, prompting urgent chest tube placement, and was confirmed on a repeat chest radiograph performed later that morning (Figure 2D). Unfortunately, the patient developed septic shock several days later and subsequently developed disseminated intravascular coagulation (DIC) before ultimately expiring on ICU day 34.

Case 2

**Figure 3:**
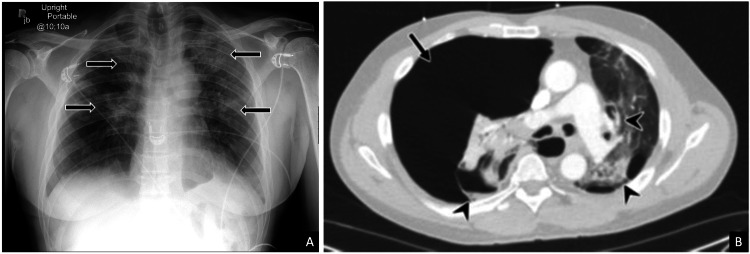
Chest radiograph and CT chest of a 61-year-old male with a recent COVID-19 infection presenting with acute-onset shortness of breath AP chest radiograph from the external hospital (A) demonstrated patchy bilateral infiltrates consistent with pneumonia (arrows), but no pneumothorax was observed. The patient was discharged on antibiotics from the hospital. However, later the same day, he presented to our hospital’s emergency department with severe shortness of breath and acute chest pain. ECG and bedside echocardiogram showed findings suggestive of right heart strain. Subsequently, STAT chest CT ordered for suspected pulmonary embolism (B) demonstrated a new large right pneumothorax (arrow) resulting in right lung collapse and contralateral mediastinal shift, representing a tension pneumothorax. Pulmonary parenchymal changes of prior COVID-19 pneumonia are also seen, including ground-glass opacities and pneumatoceles (arrowheads) AP: anteroposterior; COVID-19: coronavirus disease 2019; CT: computed tomography; ECG: electrocardiogram

**Figure 4:**
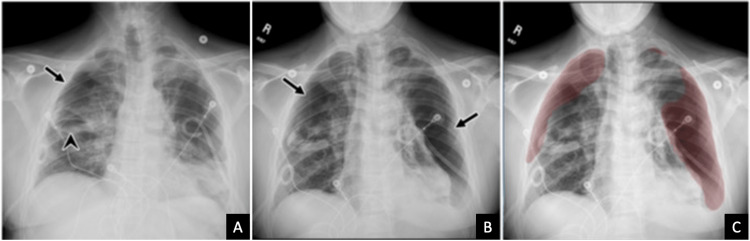
Chest radiographs of a 61-year-old male with a recent COVID-19 infection presenting with acute-onset shortness of breath Subsequent chest radiograph (A) performed the day following the initial CT scan and chest tube placement demonstrates significantly improved right pneumothorax (arrow). There is a re-expansion of the right lung with right-sided pneumatocele (arrowhead) that is now better visualized. Bilateral chest tubes are seen. Routine chest radiograph on hospital day 10 (B) demonstrates enlarging pneumothoraces (arrows) flagged by the AI algorithm (C). The findings were consistent with an air leak for which the patient underwent surgical correction AI: artificial intelligence; COVID-19: coronavirus disease 2019; CT: computed tomography

A 61-year-old male with a past medical history of asthma, emphysema, hepatitis C, and a recent diagnosis of COVID-19 two weeks prior presented to the ED with acute-onset shortness of breath. This patient had visited an external hospital earlier in the day where a chest radiograph demonstrated patchy infiltrates bilaterally, correlating to his known pneumonia secondary to a recent COVID infection (Figure 3A). He was discharged home with antibiotics but presented to the ED later that day with severe chest pain, tachypnea, and hypoxia. ECG and bedside echocardiography showed right heart strain and a STAT CT scan for suspected pulmonary embolism was ordered, which showed a new large right pneumothorax with complete collapse of the right lung, representing a tension pneumothorax that required immediate bedside chest tube placement (Figure 3B). On repeat chest radiography the following day, a new left-sided pneumothorax was discovered, prompting left-sided chest tube placement (Figure 4A). These pneumothoraces gradually improved over the next few days. However, on hospital day 10, a routine portable chest radiograph obtained on the floor was flagged in the PACS worklist by the AI tool as having a suspicious finding. A subsequent review by the radiologist demonstrated interval worsening of bilateral pneumothoraces, which was believed to be due to an air leak from ruptured pneumatoceles secondary to his recent COVID-19 infection (Figures 4B, 4C). These pneumothoraces persisted despite bilateral chest tubes, and on hospital day 12, he underwent definitive surgical correction with bilateral thoracotomies and wedge resection/blebectomies. Postoperatively, the patient's condition progressively improved over the next few days, and he was ultimately discharged home on hospital day 19.

Case 3

**Figure 5:**
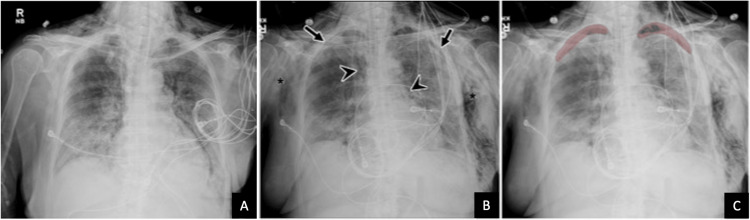
Chest radiographs of a 76-year-old female with acute hypoxic respiratory failure Initial AP chest radiograph (A) demonstrates bilateral basilar predominant, patchy airspace opacities consistent with typical COVID-19 pneumonia. Follow-up non-STAT AP radiograph (B) shows subtle small biapical pneumothoraces (arrows), pneumomediastinum (arrowheads), and extensive bilateral chest wall subcutaneous emphysema (asterisks). Pneumothoraces were correctly flagged by the AI algorithm (C) AI: artificial intelligence; AP: anteroposterior; COVID-19: coronavirus disease 2019

**Figure 6:**
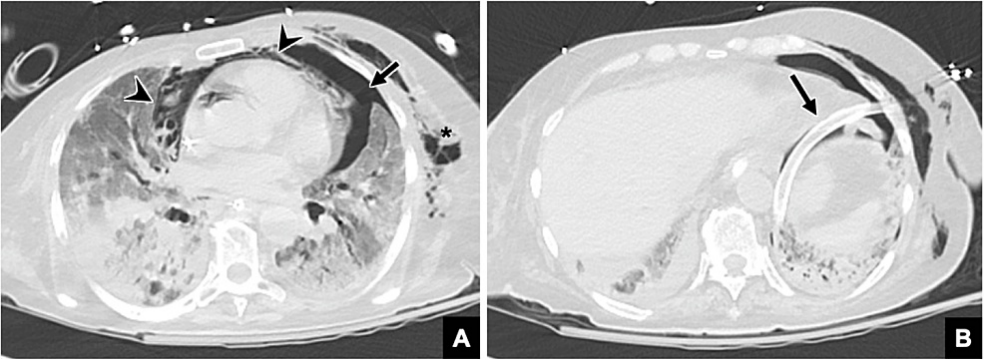
Subsequent axial CT of a 76-year-old female with acute hypoxic respiratory failure Subsequent axial CT of the chest (A, B) redemonstrates pneumomediastinum (arrowheads in A) and subcutaneous emphysema (asterisk in A) with enlarging left pneumothorax (arrow in A). A left-sided chest tube is also seen (arrow in B) CT: computed tomography

A 76-year-old female with no significant past medical history initially presented to an external hospital ED with altered mental status. She was found to have acute hypoxic respiratory failure due to COVID-19 pneumonia and required hospitalization. Her condition progressed to ARDS, requiring intubation and transfer to the ICU on hospital day 10 (Figure 5A). Over the next few days, she had minimal improvement in oxygen requirements despite multiple trials of proning. A routine portable chest radiograph performed on ICU day 12 was flagged in the PACS worklist by the AI tool as having a suspicious finding (Figures 5B, 5C). A review by the radiologist demonstrated new small subtle biapical pneumothoraces. These findings were urgently relayed to her primary team, and a chest tube was placed in a timely manner. A follow-up CT scan confirmed the full extent of barotrauma, including the enlarging left pneumothorax and pneumomediastinum (Figure 6). The patient's condition, unfortunately, continued to deteriorate, and she developed shock and became refractory to pressor support. She developed intraparenchymal and subarachnoid hemorrhage as well as retroperitoneal bleeding. After a discussion involving her family, the decision was made to pursue comfort care measures. Palliative extubation was performed on ICU day 15.

## Discussion

Established lung imaging findings reported with COVID-19 primarily pertain to CT and include multifocal ground-glass opacities in a predominately peripheral distribution with slight lower lung predilection and a striking absence of pleural effusions [6]. Chest radiographs are of limited diagnostic value early in the disease course but can be useful in detecting signs of progression, such as worsening consolidations or findings of ARDS. Although considered a relatively uncommon initial imaging finding in COVID-19, pneumothorax can also present with disease progression. It may arise either spontaneously or secondary to barotrauma and may constitute a true life-threatening emergency in these patients with pre-existing respiratory compromise. In the absence of acute changes to respiratory status or in patients on mechanical ventilation, pneumothorax may not be readily apparent on clinical exam. In such cases, the radiologist is often the first person to identify the abnormality, and timely identification by the radiologist is key to ensuring appropriate clinical care.

However, as imaging volumes continue to rise worldwide, increasing clinical demands on radiology departments have challenged current workflow models. In some settings, it may not always be feasible for radiologists to read and interpret all acquired radiographs in a timely fashion, particularly those ordered as a routine priority without clinical suspicion of an acute abnormality or those performed outside of peak radiology staff times [10]. This can lead to backlogs of unreported studies and untimely delays in the diagnosis of critical findings. Employing a reliable mechanism to screen and prioritize studies with positive or “critical” findings on the radiologist’s worklist for more rapid reviews is a welcome and necessary step toward improving radiology workflow and efficiency.

Leveraging an AI tool that expedites pneumothorax detection is highly desirable given that timely detection of moderate or large pneumothoraces can enable prompt and appropriate clinical management that mitigates the risk of serious complications. The AI tool explored in this case series, which specializes in pneumothorax detection, was first developed by researchers at the University of California, San Francisco (UCSF) and licensed to GE Healthcare for introduction into the clinical world [11]. During its development, a dataset of over 13,000 annotated chest radiographs was used to train and select the most viable models. Internal testing of the best models on a dataset of 1,701 chest radiographs yielded a receiver operator characteristic (ROC) area under the curve (AUC) of 94% [10]. Subsequently, the AI tool was integrated into GE Healthcare’s Critical Care Suite software and evaluated by the FDA for use as a triaging tool intended to alert radiologists to potentially emergent pneumothoraces.

In its evaluation by the FDA, the Critical Care Suite software was tested on a dataset of 804 frontal chest X‐rays [9]. The reads of the AI algorithm were compared to ground truth findings established by three independent US board-certified radiologists. During this evaluation, which included 376 pneumothorax-positive cases and 428 pneumothorax-negative cases, the ROC AUC was found to be 96%, which achieves the performance requirement standard for FDA product code QFM, which covers computer-assisted prioritization software for lesions. The algorithm demonstrated consistent performance across image views (i.e., anteroposterior vs. posteroanterior), system manufacturer, and data source. The AI algorithm’s specificity was 93.5% and its sensitivity was 84.3%. It successfully detected 96.3% of large pneumothoraces and 75% of small pneumothoraces with limited false-positive notifications thanks to its high specificity. Given its promise, our institution deployed Critical Care Suite in real-time clinical practice in 2020 and has continued to evaluate its accuracy and effectiveness in this role.

The three cases presented in this case series serve as a real-world examples of an AI-enabled platform in action and highlight several key benefits of this technology in clinical practice. The scenario described in the first case demonstrates the potential role of the AI-enabled tool in detecting an unsuspected pneumothorax on a routine chest radiograph, which led to the prioritization of the study’s review by the interpreting radiologist. The second case highlights the utility of the AI-enabled tool in recognizing potential complications associated with chest tube management, such as air leaks. Lastly, the third case highlights the ability of the AI-enabled tool to detect subtle pneumothoraces. Altogether, these cases illustrate how the integration of an AI-enabled portable X-ray machine may accelerate the detection and diagnosis of pneumothorax in the setting of COVID-19, thereby enabling the primary team to provide timely and appropriate treatment to minimize complications from pneumothorax in already critically ill patients.

While our case series documents real-world examples of one particular AI tool's utility in assisting and expediting the detection of pneumothoraces in ICU patients, the potential for continually advancing AI to be leveraged more broadly within intensive care medicine represents an exciting prospect for the future. A few possible uses that have been explored include predicting a patient’s length of stay in the ICU, evaluating a patient’s likelihood of developing complications, and assisting in fine-tuning mechanical ventilator parameters for a given patient [12,13]. However, significant challenges are associated not only with the technical development of such tools but also their validation in real-world clinical settings. In addition to these clinician-oriented applications of AI, there is also potential for imaging-oriented applications similar to the one presented in our case series. One such AI tool that has been developed aims to automate the evaluation of endotracheal (ET) tube placement, which has traditionally been assessed through the manual interpretation of chest radiographs [14]. Deployment of this AI tool in real-world clinical practice is expected to expedite the detection of faulty ET tube placements and thus mitigate associated complications to improve ICU patient outcomes. Continual investigations into how AI can be beneficially integrated into clinical practice will be an important area of study to help usher medicine into a future where AI is ceaselessly growing in prominence.

## Conclusions

Pneumothorax is an uncommon but possible complication in critically ill patients with COVID-19 infection. The interpreting radiologist is often the first person to identify this abnormality on routine chest radiographs and hence has the potential to add significant clinical value in managing this patient population through timely and accurate interpretation. The timeliness of interpretation of these critical findings relies in large part on the acuity of the order requisition and worklist demands of the radiologist. This report described a few cases that illustrated how the employment of AI-enabled software capable of analyzing and prioritizing studies in the radiologist’s worklist can potentially lead to earlier detection of emergent findings like pneumothorax, thereby greatly improving radiology workflow and ultimately patient outcomes.
